# Tight Lacing

**Published:** 1882-11

**Authors:** 


### TIGHT LACING.

WE enter upon the discussion of this question with considerable reluctance. It is a subject the mere mention of which is "tabooed" in polite society. He who is bold enough to protest against the prevailing fashions, whether of corsets, banged hair or high heels, must be prepared to encounter the frowns of the fairest, and, perhaps, excommunication from all social circles in which he dare exercise the liberty of free speech. It is utterly useless to attempt to convince young women of the evils that later come from lacing. No child is satisfied that fire will burn until his own fingers have been blistered, and, strange as it may seem, it is quite as difficult to convince matrons of middle age when they have ha l their forms from childhood held in the grip of steel and buckram. Why don't the doctors mind their own business ? says madam, as she hauls on the cords that distort Nature's perfect work.

"Were I to leave off my corsets," says she, "I should be limp as a rag. These strengthen and sustain me." Quite right, madam; but why ? Simply because you have worn corsets so long that they have appropriated the office that the muscles of the chest were intended for, and these, having nothing to do, have dwindled away or perished, leaving the upper half of your body to be supported by corsets; just as a person who has lost a leg hobbles through life with a wooden one. What Nature has no use for she finally dispenses with. She is generous, but at the same time exercises a wise economy and does not long burden us with useless gifts. Paralysis of the pectoral muscles is the least of the evils induced by lacing. Long continued pressure on the vital organs impedes their action and deranges their functions. It is a prominent cause of heart disease, consumption and spinal irritation; and then it is not pleasant to think that the hour-glass waist suggests adhesions between the vital organs and the lining membrane of the chest. It is not necessary to be a physiologist to feel a sort of disgust for an abnormally small waist, or a philanthropist to pity its victim. There is something barbarous and repulsive in the fashion of making cripples of Chinese women by lacing their feet. Would it be more humane to make perpetual invalids of them by lacing their bodies?

